# A Broad-Range Disposable Electrochemical Biosensor Based on Screen-Printed Carbon Electrodes for Detection of Human Noroviruses

**DOI:** 10.3389/fbioe.2022.845660

**Published:** 2022-03-18

**Authors:** Nan Wang, Guiying Pan, Shimin Guan, Shaofeng Rong, Dapeng Wang, Zhiyong Gao, Peng Tian, Qianqian Li

**Affiliations:** ^1^ Department of Bioengineering, Shanghai Institute of Technology, Shanghai, China; ^2^ Department of Food Science and Technology, School of Agriculture and Biology, Shanghai Jiao Tong University, Shanghai, China; ^3^ Beijing Center for Disease Prevention and Control, Beijing Research Center for Preventive Medicine, Beijing, China; ^4^ Produce Safety and Microbiology Research Unit, Western Regional Research Center, Agricultural Research Service-United States Department of Agriculture, Albany, CA, United States

**Keywords:** human norovirus, electrochemical biosensor, viral capsid proteins, screen-printed carbon electrode, AuNPs, protein-A

## Abstract

Human noroviruses (HuNoVs) are the major non-bacterial pathogens causing acute gastroenteritis in people of all ages worldwide. No stable culture system *in vitro* is available for routing the detection of multiple strains of HuNoVs. A simple and rapid method for detection of HuNoVs is of great significance for preventing and controlling this pathogen. In this work, an electrochemical biosensor for sensitive and fast detection of HuNoVs was constructed based on a screen-printed carbon electrode (SPCE). Gold nanoparticles and protein-A were applied on the SPCE surface for enhancement of the electrical signals and the linkage of antibodies with a fixed orientation, respectively. A monoclonal antibody (MAb) against the S domain protein of the viral capsid (VP1) was further immobilized on the SPCE to bind HuNoVs specifically. The binding of VP1 to the coated MAbs resulted in the reduction of conductivity (current) measured by cyclic voltammetry (CV) and differential pulse voltammetry (DPV). The reduction in the current was correlated to the concentration of VP1/HuNoVs. The detection limitation of Genogroup I.1 (GI.1) VP1 and Genogroup II.4 (GII.4) VP1 was 0.37 ng/ml (≈1.93×10^7^ HuNoVs/mL) and 0.22 ng/ml (≈1.15×10^7^ HuNoVs/mL), respectively. The detection limitation of both GI and GII HuNoVs in clinical fecal samples was 10^4^ genomic copies/mL. The results could be obtained in 1 h. We demonstrated that this disposable electrochemical biosensor was a good candidate for rapid detection of different genogroup and genotype HuNoVs.

## 1 Introduction

Noroviruses (NoVs) are non-enveloped, single-stranded positive-sense RNA viruses of the Caliciviridae family (Jiang et al., 1993). Except for murine norovirus, the RNA genome of noroviruses is divided into three open reading frames (ORFs) (McFadden et al., 2011). ORF1 codes for non-structural viral proteins, ORF2 codes the major viral capsid protein (VP1) which consists of highly variable protruding domains (P1 and P2 sub-domains) and extremely conservative shell domain (S), and ORF3 codes the minor viral capsid protein (VP2). The viral capsid is composed of 90 VP1 dimers and some VP2 (Tan et al., 2008). NoVs have been classified into ten genogroups (GI-GX) and further divided into 48 confirmed capsid genotypes based on amino acids of the VP1, among which GI, GII, GIV, GVIII, and GX can infect humans and are usually called human noroviruses (HuNoVs) (Chhabra et al., 2019; Zheng et al., 2006). HuNoVs are the primary non-bacterial pathogens causing acute gastroenteritis and were listed on the top of the list of foodborne pathogens by the WHO (Ahmed et al., 2014; World Health Organization, 2015). The ability to rapidly detect and identify HuNoVs is extremely important to maintain public health safety (Vinjé, 2015).

Conventional technologies for HuNoV diagnostics are mainly divided into immunological assays (such as ELISA and ICG) and molecular assays (such as RT-qPCR and RT-LAMP). RT-qPCR is the gold standard for the detection of HuNoVs because of its excellent sensitivity and specificity, but it requires time-consuming sample pretreatment, long analysis time, skilled technicians, and expensive laboratory facilities. The development of widely reactive immunological assays remains challenging due to the large number of antigenically distinct HuNoV genotypes and unpredictable mutations at the antigen site in the P domain of VP1 over time (Hutson et al., 2004; Vinjé, 2015). It has been reported that the S domain of VP1 is highly conserved in HuNoVs (Miyazaki et al., 2015). A broad cross-reactive epitope was identified in the S domain of VP1 (Parra et al., 2013). It was also found that the monoclonal antibody (MAb) against the S domain of VP1 could cross-react with GI, GII, GIII, and GV of HuNoVs (Li et al., 2010). Therefore, the S domain protein could be used as a good target in immunological assays. In this study, we applied an S domain protein-specific monoclonal antibody (MAb) to develop a sensitive, fast, and portable device for the detection of multiple strains of HuNoVs from different genogroups.

Electrochemical biosensors are considered as reliable analytical devices and represent a prospective tool for the detection of different pathogenic viruses (Khan et al., 2020). Many sensors have been reported to be applied in pathogen detection (Wang et al., 2009; Ilkhani and Farhad, 2018; Shi et al., 2020). To date, there were a few reports of electrochemical biosensors for HuNoV detection. Hong et al. (2015) proposed an electrochemical biosensor that used concanavalin A as a recognition element for the selective capture of NoV and affinity peptide was employed to identify NoV (Hwang et al., 2017). However, only a couple of genotypes of HuNoVs could be detected by those biosensors (Wang et al., 2021). It is crucial to develop an assay for the detection of multiple genotypes of HuNoVs with high sensitivity and selectivity.

Screen-printed electrodes are excellent electrochemical sensor elements that do not require cumbersome pretreatment (such as polishing) and with merits of ability for mass production, low cost, small size, flexible design, and easily integration with other equipment (such as microfluidic device) (Alonso-Lomillo et al., 2010; Chen et al., 2019; Devi and Krishnan, 2020). Gold nanoparticles (AuNPs) are usually engaged in electrochemical biosensors to amplify signals, owing to their excellent electrical properties, large specific surface area, and biocompatibility and binding ability to a range of biomolecules (Fei et al., 2015; Florea et al., 2013). Staphylococcal protein-A (protein A) can be adsorbed on the surface of gold nanoparticles through hydrophobic interaction (Hermanson, 2008). It has been proved that antibody-binding proteins have a high affinity for the Fc region of some antibodies (especially IgG) (Aybay, 2003; Rigi et al., 2019). This mode of immobilization results in a uniform orientation of the antigen-binding sites in the Fab fragment which maximizes the antigen-binding capability of the antibody. In this study, we applied this strategy to develop an electrochemical biosensor-based point-of-care testing (POCT) for the detection of HuNoVs. The developed screen-printed carbon electrode (SPCE) contains AuNPs, protein-A of *Staphylococcus*, and an anti-S domain MAb (H9E). Cyclic voltammetry (CV), differential pulse voltammetry (DPV), and electrochemical impedance spectroscopy (EIS) were used to analyze the interaction between the antibody and antigen. To the best of our knowledge, this is the first time that the anti-S-domain antibody has been used in the preparation of electrochemical biosensors for the detection of different genogroup and genotype HuNoVs.

## 2 Materials and Methods

### 2.1 Reagents and Apparatus

Tetrachloroauric (III) acid tetrahydrate (HAuCl_4_·4H_2_O) was purchased from Sinopharm Chemical Reagent Co., Ltd. (Shanghai, China). Potassium hexacyanoferrate (III) (K_3_[Fe(CN)_6_]) and potassium hexacyanoferrate (II) trihydrate (K_4_[Fe(CN)_6_])·3H_2_O) were purchased from Aladdin Biochemical Technology Co., Ltd. (Shanghai, China). Recombinant Staphylococcal protein-A was purchased from NeuroPeptide Biotech Co., Ltd. (Hangzhou, China). The Bradford Protein Assay Kit was purchased from Beyotime Biotech Co., Ltd. (Shanghai, China). Bovine serum albumin (BSA) was purchased from Yeason Biotech Co., Ltd. (Shanghai, China). pET28a-ORF2 (GI.1) and pET28a-ORF2 (GII.4) encoding VP1 were constructed as described previously (Wang et al., 2017). All chemicals were of analytical grade and were used without further purification. All solutions were prepared with deionized water purified through a Milli-Q system.

**FIGURE 1:**
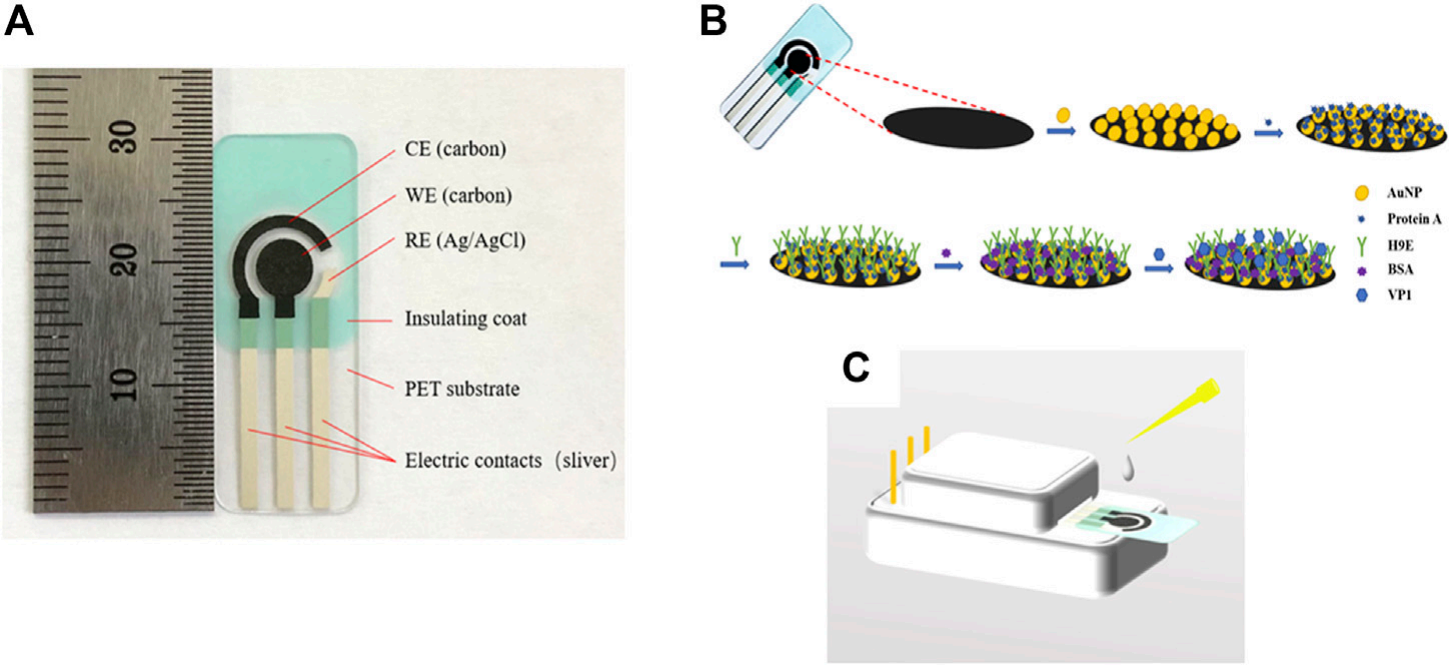
Profile of the screen-printed carbon electrode **(A)**. Schematic representation of the stepwise modification of electrochemical biosensor **(B)** and measurement element **(C)**. CE: counter electrode, WE: wording electrode, RE: reference electrode, AuNPs: gold nanoparticles, protein A: Staphylococcal protein-A, H9E: monoclonal antibody generated by recombinant capsid proteins of norovirus, BSA: bovine serum albumin, VP1: major viral capsid protein.

All the electrochemical measurements were performed using a CHI760D electrochemical workstation (Chenhua Instruments, Inc. Shanghai, China). The SPCEs were purchased from Qingdao Poten Technology Co., Ltd. (Qingdao, China). The SPCE has a traditional three-electrode configuration, which comprises a working electrode (WE) (5 mm in diameter), a counter electrode (CE), and a reference electrode (RE) (Figure 1A). The surface structure of the working electrode was characterized using a Hitachi SU8010 (company info.) field emission scanning electron microscope (FE-SEM).

### 2.2 Preparation of Clinical Fecal Samples

Three clinical fecal samples from patients infected with GII.4 or GI.1 HuNoVs were provided by Prof. Dapeng Wang (Shanghai Jiao Tong University). The use of fecal samples was approved by the Institutional Bio-safety Committees (IBC) of the College of Agriculture and Biology, Shanghai Jiao Tong University. The three samples numbered 1717, 1704, and 3010 were represented as GII.4, GII.4 and GI.1, respectively. The viruses were quantified by RT-qPCR as described previously. The genomic copies of 1717, 1704 and 3010 were 4.4×10^5^, 1.31×10^9^, and 3.24×10^6^, respectively (Xu et al., 2021). The viral capsids including the S domain in these fecal samples were exposed by heating at 80 °C for 3 min. The genomic copies of the viruses were quantified by RT-qPCR as described previously (Xu et al., 2021). The viral capsids including the S domain in these fecal samples (10.0% (w/v) fecal sample diluted with PBS) were exposed by heating at 80 °C for 3 min. The samples were clarified by centrifugation at 10,000×g for 10 min and then stored at -20 °C for further use as described previously (Xu et al., 2021).

### 2.3 Preparation of Recombinant Capsid Proteins

Recombinant capsid proteins (rVP1s of GI.1 and GII.4) were expressed and purified as described previously (Niu et al., 2015). The rVP1s were analyzed using the SDS-PAGE according to the previous report (Wang et al., 2008). The concentration of rVP1 protein was quantified using a commercial Bradford assay kit. The purified rVP1s were then stored at −20°C for further use. The MAb (H9E) was generated with VP1 and had the ability to bind the S domain of VP1s from both GI and GII HuNoVs (Xu et al., 2021).

### 2.4 Preparation of Electrochemical Biosensors

The SPCE was electrochemically pretreated by CV in the potential range of −1.5 to 1.5 V and a scan rate of 0.1 V/s for 10 cycles with 0.5 M H_2_SO_4_ solution (Goud et al., 2017). The electrode was then thoroughly rinsed with deionized water. After these pretreatments, 100 µL of HAuCl_4_·4H_2_O solution (0.1%, w/v) was dropped onto the electrode surface and electrodeposited under the deposition potential of −0.2 V for 240 s (denoted as AuNPs/SPCE). Next, 10 µL protein-A (10 μg/ml) was dropped onto the working electrode surface, incubated at 37°C for 30 min, rinsed with deionized water, and dried at room temperature (denoted as protein-A/AuNPs/SPCE). Subsequently, the electrode was incubated with 10 µL MAb H9E (0.1 mg/ml) for further 45 min at 37 °C (denoted as H9E/protein-A/AuNPs/SPCE). After rinsing with PBS-T (PBS containing 0.05% Tween-20, pH 7.4) followed by deionized water and drying at room temperature, 10 µL BSA (1%, w/v) was incubated on the electrode surface at 37 °C for 30 min to block non-specific binding sites (denoted as BSA/H9E/protein-A/AuNPs/SPCE). Finally, the biosensor (BSA/H9E/protein-A/AuNPs/SPCE) was stored at 4 °C for further use after rinsing with PBS followed by deionized water. All biological modification of electrodes was performed in a wet box. The FE-SEM images of SPCE and AuNPs/SPCE were obtained with a scale bar of 1.0 μm. The diameter distribution of AuNPs was measured by Nano Measurer 1.2 software.

### 2.5 Electrochemical Measurements

The schematic representation of the biosensor was depicted (Figure 1B). All electrochemical measurements were carried out by placing 100 µL of electrolyte onto the three electrode areas (shown in Figure 1C). The samples detection procedure was as follows: 10 µL sample solution was dropped onto the previously modified electrode, incubated at 37°C for 45 min, rinsed carefully with PBS, and deionized with water to remove the unbound antigen. CV and DPV were carried out in 0.1 M KNO_3_ (as the supporting electrolyte) solution containing 5 mM [Fe(CN)_6_]^3-/4-^ (1:1). The EIS measurement was carried out in 0.01 M PBS (pH 7.4) containing 5 mM [Fe(CN)_6_]^3-/4-^ (1:1) and 0.1 M KCl (as the supporting electrolyte). CV measurements were recorded in the potential range of −0.8 to 1.0 V at a scan rate of 50 mV/s (Goud et al., 2017; Hayat et al., 2012). The DPV experiment was studied in a positive range of -0.2 to 0.6 V (Goud et al., 2017; Hayat et al., 2012). The detection of the rVP1/clinical samples was evaluated by measuring the relative peak current decrease of DPV before (I_0_) and after (I) exposing to rVP1/clinical samples. The relative peak current decrease was calculated as follows:

$$\%\;decreasing\;current\;=\frac{I-I_{0}}{I_{0}}\times 100\%.$$

EIS analysis was performed at open circuit potential with an oscillation of 5 mV amplitude over the frequencies from 1 to 100 kHz with a sampling rate of 12 points per ten times the frequency (Goud et al., 2017). The collected EIS was fitted in the theoretical equivalent of modified Randles equivalent circuit using ZView software. All measurements were performed at room temperature.

### 2.6 Reproducibility, Selectivity, and Stability Analysis

Seven electrochemical biosensors were selected randomly. The GII.4 rVP1 with a concentration of 10 ng/ml was used to evaluate the reproducibility.

Murine norovirus (MNV) and Tulane virus (TV) were used to test the specificity of the biosensor. TV and MNV were heat-denatured and were one hundred-fold diluted by PBS as described previously (Xu et al., 2021). MNV and TV lysates without adding GII.4 rVP1 were used as negative controls. In addition, MNV and TV lysates or both were mixed with 10 ng/ml of GII.4 rVP1 at a ratio of 1:1 (v:v) to test their influence on VP1 binding on the biosensor. DPV was used to detect and analyze the specificity.

Electrochemical biosensors were sealed and preserved at 4°C for 2 weeks with a measurement for every 2-day interval. The GII.4 rVP1 with a concentration of 10 ng/ml was used to analyze the stability.

Electrochemical measurements were performed as described in Section 2.5. For stability studies, the current decreasing percentage of the first detection day was considered 100%. Each experiment was repeated three times as independent replicates. The relative standard deviation (RSD) was calculated (Upan et al., 2020). One-way ANOVA was employed for data analysis. *p* < 0.05 was considered significant.

## 3 Results and Discussion

### 3.1 Characterization of the Electrochemical Changes in Each Step in Building Up the Biosensor

**FIGURE 2:**
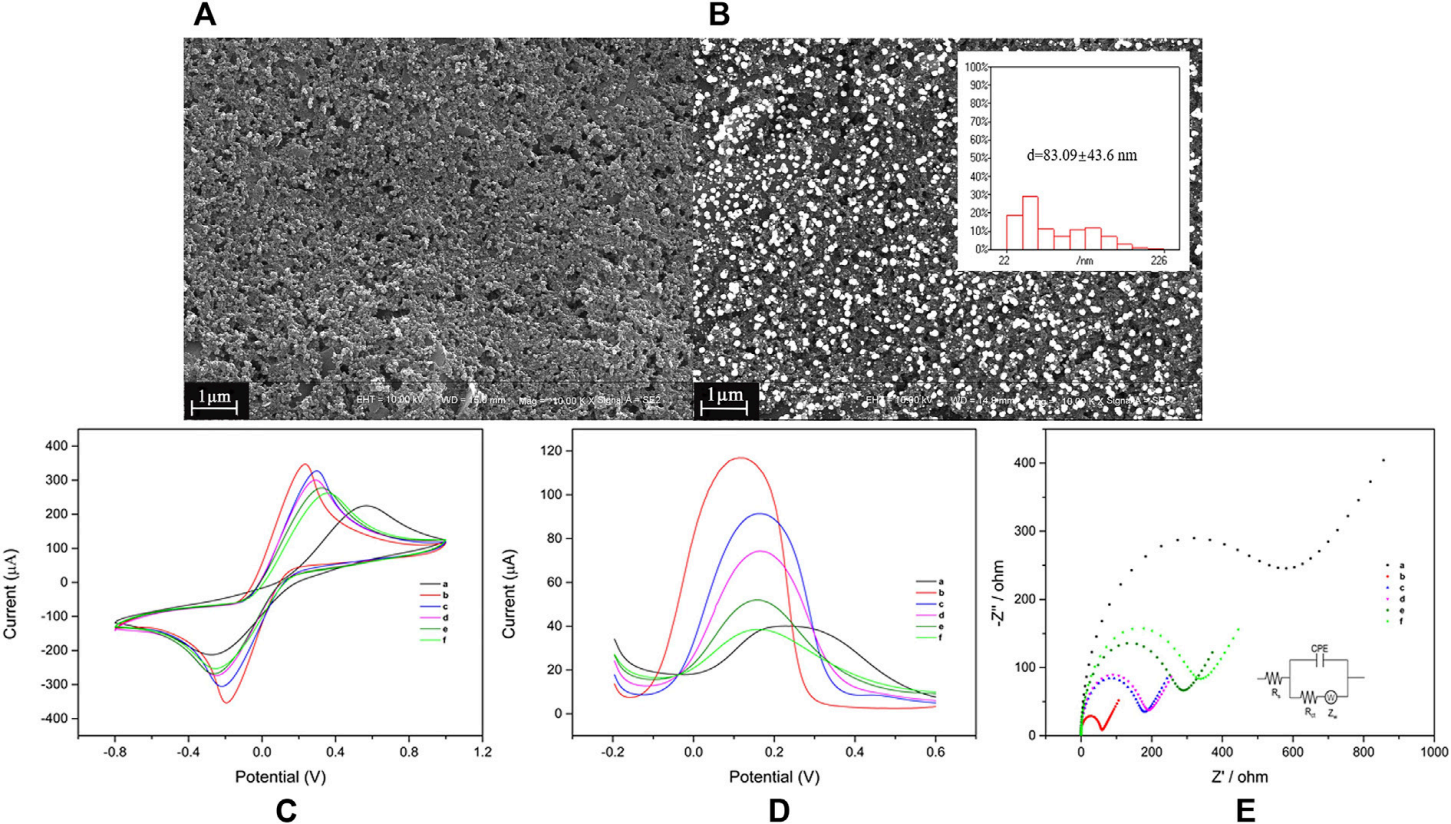
Preparation and characterization of the electrochemical biosensor for norovirus detection. Field emission scanning electron microscope images of SPCE **(A)** and AuNPs/SPCE **(B)**, scale bar is 1.0 μm. The AuNP diameter (**B**, inset), cyclic voltammetry **(C)**, differential pulse voltammetry **(D)**, and electrochemical impedance spectroscopy **(E)**; measurements of SPCE (a), AuNPs/SPCE (b), protein-A/AuNPs/SPCE (c), H9E/protein-A/AuNPs/SPCE (d), BSA/H9E/protein-A/AuNPs/SPCE (e), and VP1/BSA/H9E/protein-A/AuNPs/SPCE (f). The diagram of the modified Randles equivalent circuit (E, inset). The concentration of GII.4 rVP1 was 10 μg/ml. SPCE: screen-printed carbon electrode, AuNPs: gold nanoparticles, protein A: Staphylococcal protein-A, H9E: monoclonal antibody generated by recombinant capsid proteins of norovirus, BSA: bovine serum albumin, VP1: major viral capsid protein.

The modification process of electrode was characterized by FE-SEM, CV, DPV, and EIS. FE-SEM was first used to study the surface morphologies of the electrode that electrodeposited with AuNPs. Figure 2A shows the FE-SEM image of SPCE, and Figure 2B shows clearly that relatively uniform AuNPs were attached to the surface of AuNPs/SPCE. The diameter of AuNPs was 83.09 ± 43.6 nm with a range from 22.68 to 225.83 nm (Figure 2B inset).

Each modification step of the biosensor was optimized (Supplementary Figure S1) and evaluated. When AuNPs were deposited on the surface of SPCE (Figure 2C,D; curve a), the peak current was increased remarkably (Figure 2C,D; curve 2) due to the excellent electron transfer performance of AuNPs. The presence of AuNPs increased the specific surface area of the electrode and the active sites for electron transfer (Fei et al., 2015). The shift of maximum potential peak was due to the nature of AuNPs (Munteanu and Apetrei, 2021). Then, protein-A was immobilized to increase the affinity for the Fc region of H9E. BSA was further used to avoid the unspecific binding to the biosensor. Subsequently, along with the incorporation of protein-A, H9E, and BSA onto AuNPs/SPCE, the peak current was reduced accordingly (Figure 2C,D; curves c to e) as expected. It can attribute to the fact that biomolecules hinder the electron transfer toward the electrode surface. Finally, the binding of VP1 to the MAbs on the biosensor resulted in a significant reduction of peak currents (change from Figure 2D curves e to f) as expected.

EIS was also used to investigate the modification situation of the electrode. The collected data were fitted in a modified Randles equivalent circuit (Figure 2E; inset). The typical Nyquist plot includes the semicircle part (high frequency) and linear part (low frequency). The semicircle diameter of the high-frequency portion corresponds to the interface electron transfer resistance (R_ct_) between the electrode and electrolyte solution (Goud et al., 2017; Qiu et al., 2016). The modification of the electrode surface affects the interface’s electronic transfer dynamics, which changes the electron transfer resistance (Tang et al., 2007). As shown in Figure 2E, the SPCE modified with AuNPs displayed a smaller diameter (curve b, 56.3 Ω) contrasted to the bare SPCE (curve a, 506.3 Ω) and excellent conductivity. Following that, after the incorporation of protein-A (curve c, 162.3 Ω), H9E (curve d, 172.1 Ω), and BSA (curve e, 258.5 Ω), the semicircle diameter exhibited a distinct successive increase, which was ascribed to the biomolecules hindering the electron transfer process of the electrode–solution interface. Similarly, the R_ct_ values were increased to 298.5 Ω (curve f) after interacting with both the antibody (H9E) and antigen (VP1). These results confirmed that all the materials were successfully modified to the electrode surface, and the biosensor (BSA/H9E/protein-A/AuNPs/SPCE) could be used to detect VP1.

**FIGURE 3:**
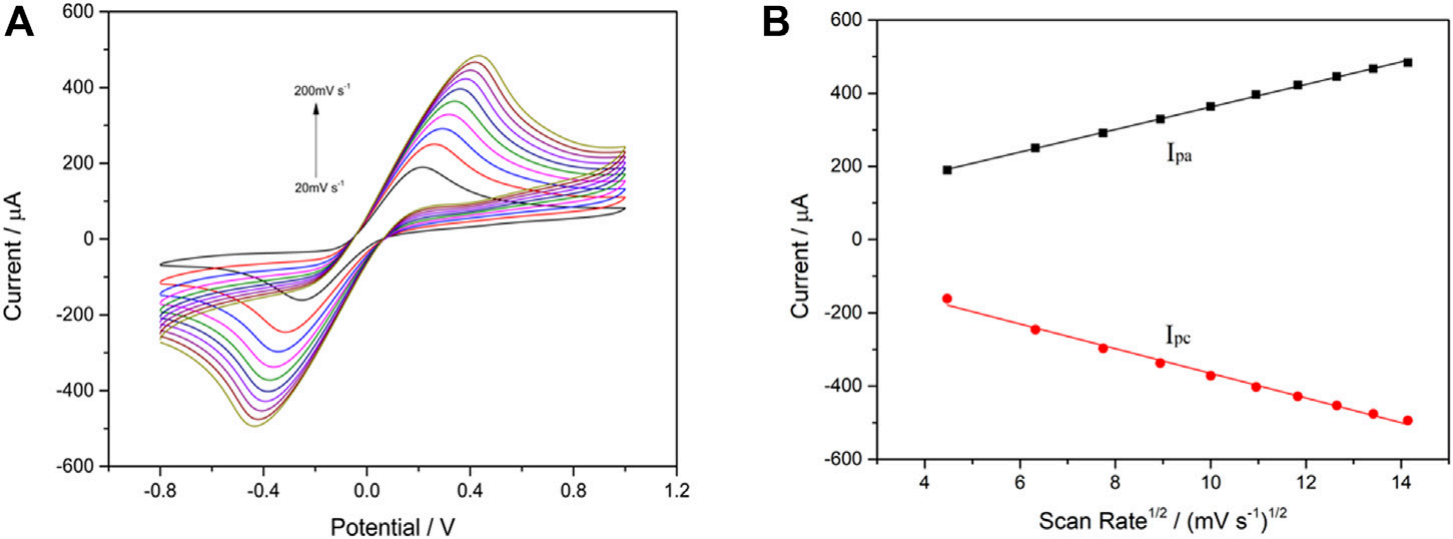
Cyclic voltammetry performance of VP1/BSA/H9E/protein-A/AuNPs/SPCE in 5.0 mM [Fe(CN)_6_] ^3-/4-^ (1:1) and 0.1 M KNO_3_ at different scan rates (20–200 mV s^−1^) **(A)**. Linear relationship between scan rate^1/2^ and peak current **(B)**. The black line and red line are I_pa_ and I_pc_, respectively. Linear regression equation: I_pa_ (µA) = 54.39 + 30.81v^1/2^ ((mV s^−1^)^1/2^; *R* ^2^ = 0.9988); I_pc_ (µA) = -27.84–33.72v^1/2^ ((mV s^−1^)^1/2^; *R* ^2^ = 0.9925).

Furthermore, the correlation between the cathodic/anodic peak current and scan rate was evaluated in each step during building up the VP1/BSA/H9E/protein-A/AuNPs/SPCE biosensor. The CV measurements were carried out in 0.1 M KNO_3_ solution containing 5 mM [Fe(CN)_6_]^3-/4-^ (1:1) under different scan rates (20–200 mV s^−1^). As shown in Figure 3A, the cathodic peak current (I_pc_) and anodic peak current (I_pa_) were changed along with the scan rates. Furthermore, it was found that the I_pc_ and I_pa_ values were in a good linear relationship with the square root of scanning rates (v^1/2^) (Figure 3B). The results suggested that the electrochemical process of the interface for the biosensor was controlled by diffusion (Munteanu and Apetrei, 2021). The current response signal generated by this biosensor was only related to the concentration of the tested antigen. Therefore, the biosensor had a good electrochemical performance.

### 3.2 Analytical Performance of the Biosensor for Detection of Recombinant GI.1 rVP1 and GII.4 rVP1

**FIGURE 4:**
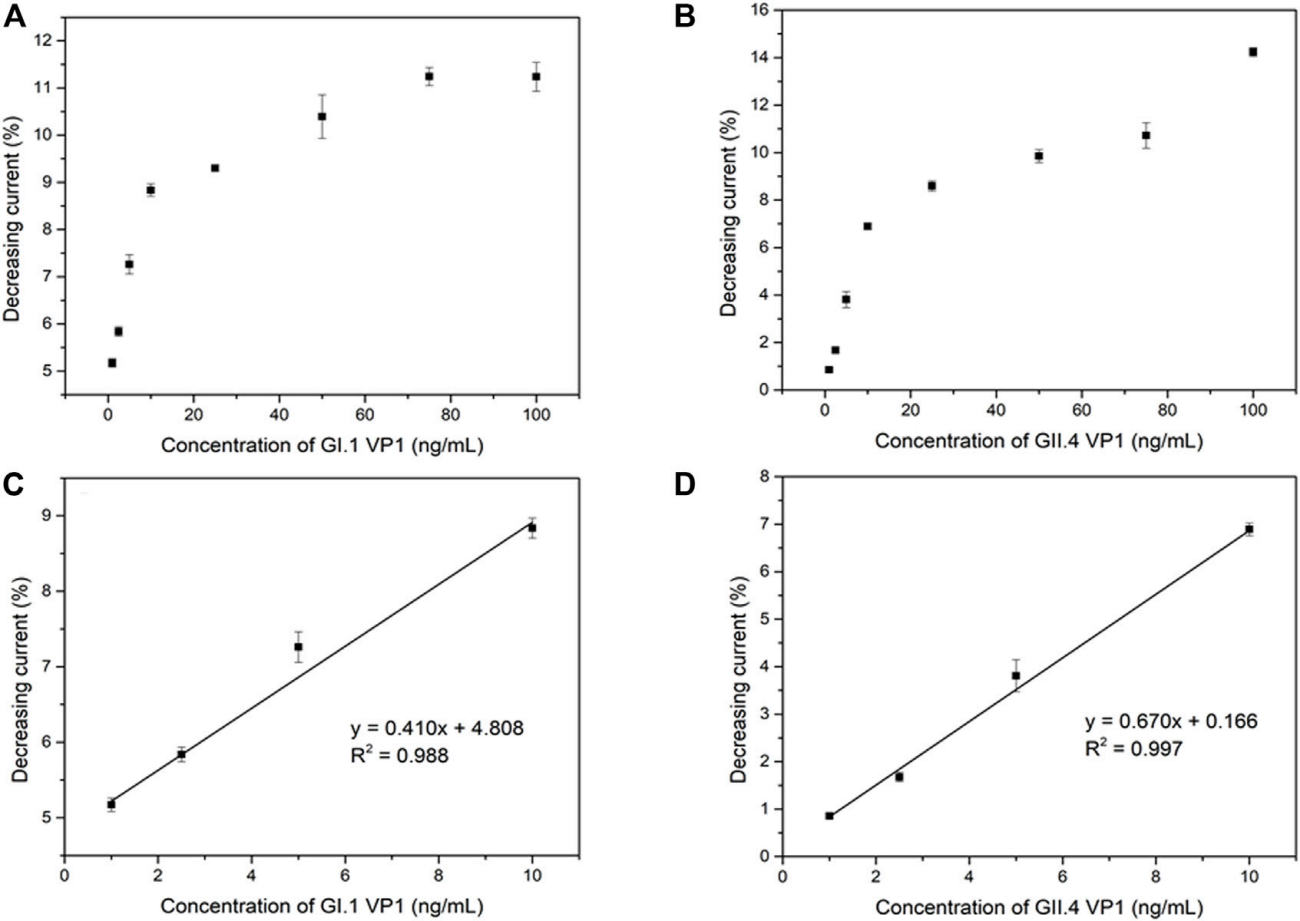
Correlation between shifting current percentage and the GI.1 rVP1 concentration **(A)**, GII.4 rVP1 concentration **(B)**, and corresponding calibration curve **(C) (D)**.

The analytical performance of the electrochemical biosensor for the detection of HuNoVs GI.1 rVP1 and GII.4 rVP1 was measured by DPV. The binding of rVP1s to MAb on the biosensor is expected to cause an increase in resistance and a reduced current. As expected, DPV peak currents were inversely proportional to the exposed concentrations of both GI.1 rVP1 and GII.4 rVP1. Figure 4A,B shows the relationship between the decreasing peak current (in percentage) and GI.1 rVP1 and GII.4 rVP1 concentrations ranging from 1 to 100 ng/ml, respectively. The corresponding calibration curve for GI.1 rVP1 and GII.4 rVP1 is shown in Figure 4C,D. The limits of detection (LOD) were calculated based on 3σ/slope (Kang et al., 2021). The calculated LODs were 0.37 ng/ml and 0.22 ng/ml for GI.1 rVP1 and GII.4 rVP1, respectively. The results demonstrated that the proposed biosensor could be applied to the diagnosis of both GI.1 and GII.4 HuNoVs. As the S domain of VP1 is highly conserved among HuNoVs, the assay has the potential to be applied to detect other HuNoVs from different genogroups and genotypes.

### 3.3 Reproducibility, Selectivity, and Stability of the Biosensor

**FIGURE 5:**
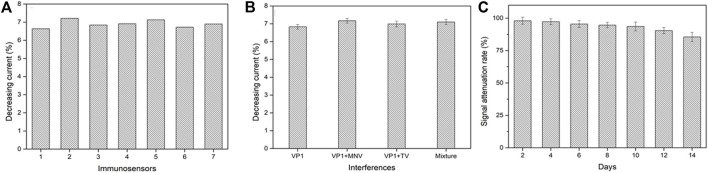
Reproducibility **(A)**, selectivity **(B)**, and stability **(C)** of the electrochemical biosensor. VP1, GII.4 rVP1; VP1+MNV, GII.4 rVP1 mixed with murine norovirus lysates; VP1+TV, GII.4 rVP1 mixed with Tulane virus lysates; mixture, GII.4 rVP1 mixed with both murine norovirus and Tulane virus lysates.

The reproducibility, selectivity, and stability are critical indicators to evaluate the performance of an electrochemical biosensor. Under the same experimental conditions, seven biosensors were randomly selected for the detection of GII.4 rVP1. The average decreasing current percentage was 6.91% (Figure 5A). The results showed 2.97% relative standard deviation (RSD), which exhibited good reproducibility.

To assess the specificity of a biosensor, the GII.4 rVP1 (10 ng/ml) with the presence of potential interference was used to test at 1:1 (v: v), including TV lysate and MNV lysate. As shown in Figure 5B, the decreasing relative peak current had no significant difference in current signals when GII.4 rVP1 was applied to the biosensor in the presence or absence of other interferences (TV and MNV) (*p* > 0.05). It was further confirmed that no decrease in current was detected when biosensors were tested with TV lysate or MNV lysate, indicating that a reduction in current in the biosensor was specific for HuNoVs. These results demonstrated that the electrochemical biosensor had a good specificity and could be used to detect HuNoV VP1 effectively. The excellent selectivity of this biosensor is mainly benefited by the high specificity of monoclonal antibodies.

The stability of the biosensor was evaluated by detecting the current signal of 10 ng/ml GII.4 VP1 after being hermetically stored in the refrigerator at 4 °C for 2 weeks (recorded the biosensor activity every 2 days). As shown in Figure 5C, compared to the initial current, the signal attenuated about 14.5% after stored for 2 weeks. The results showed that the biosensor had acceptable stability.

### 3.4 LODs of the Biosensor in Detection of HuNoVs in Clinical Fecal Samples

**FIGURE 6:**
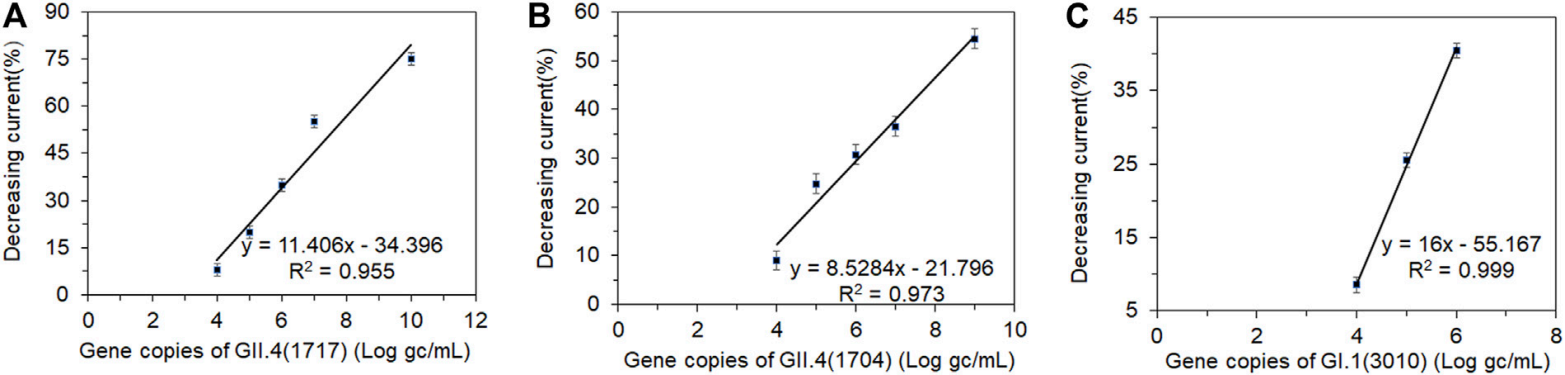
Correlation between the shifting current percentage and a series of dilutions (log gc/L) of clinical fecal samples. Human norovirus samples **(A)** GII.4 (1717), **(B)** GII.4 (1704), and **(C)** GI.1 (3010). gc, genomic copies.

There was a good correlation between the currents and viral loads (Figure 6). The original concentrations of the clinical fecal samples HuNoVs GII.4 (1717), GII.4 (1704), and GI.1 (3010) were 3.48×10^10^, 1.31×10^9^, and 3.24×10^6^ genomic copies/mL, respectively (Xu et al., 2021). The current was measured in serially diluted viral samples ranging from 10^7^ to 10^2^ genomic copies/mL. The peak current detected by DPV was inversely proportional to the concentrations of GI.1(3010) and GII.4 (1717 and 1704). Figures 6A–C shows the relationship between the decreasing peak current percentage and different concentrations of GI.1 and GII.4, respectively. A significant decrease in current was detectable at a viral load ranging from 10^4^ to 10^7^ genomic copies/mL. The decrease in current was undetectable when viral loads were between 10^2^ and 10^3^ genomic copies/mL. This finding indicated that the LOD of this biosensor for GI.1 and GII.4 virus samples was about 10^4^ genomic copies/mL. The effective testing for GI and GII HuNoV clinical samples demonstrated that this biosensor had potential use in different genogroup and genotype HuNoV testing. Xu et al. (2021) reported that the LOD of a colloidal gold-based immunochromatographic assay (ICA) was 10^6^ and 10^5^ in clinical samples for GI and GII HuNoVs, respectively. The electrochemical biosensors which we had constructed improved the sensitivity by a log for GII and two logs for GI. The electrochemical biosensor should be sensitive enough for most clinical samples which usually contain much more viruses than the LOD. This biosensor could be further improved for environmental and food samples which contain much less viral load.

## 4 Conclusion

We established an electrochemical biosensor that targeted HuNoV shell domain proteins and tested the ability to detect the rVP1 of both GII.4 and GI.1, and HuNoVs from clinical fecal samples. AuNPs were used for electrical signal enhancement, and protein-A was used to link antibodies in a fixed direction. The DPV results showed that the LOD for GI.1 rVP1 was 0.37 ng/ml and the LOD for GII.4 rVP1 was 0.22 ng/ml. The LOD of the clinical fecal samples was 10^4^ genomic copies/mL for both GI and GII HuNoVs. The total time of HuNoV VP1 detection was less than 1 h (from sample to result). Moreover, this study is the first report using a monoclonal antibody against the shell domain proteins of HuNoVs in the preparation of electrochemical biosensors, which have the potential to be used to detect different genogroup and genotype HuNoVs. Future research should focus on optimizing the parameters of electrochemical biosensors and using them for clinical and food sample testing to optimize and evaluate the suitability and accuracy of the electrochemical biosensors.

## Data Availability

The original contributions presented in the study are included in the article/Supplementary Materials, further inquiries can be directed to the corresponding author.
