# Supplementary material for: A Broad-Range Disposable Electrochemical Biosensor Based on Screen-Printed Carbon Electrodes for Detection of Human Noroviruses
Source: Front Bioeng Biotechnol. 2022 Mar 18;10:845660. doi: 10.3389/fbioe.2022.845660 (PMC8992780; doi:10.3389/fbioe.2022.845660)
Supplement: Supplementary file 1 [file DataSheet1.PDF]

In order to obtain the best working performance of the biosensor, the electrodeposition time of gold nanoparticles, protein A immobilization time, antibody and antigen incubation times were optimized. According to the results, the optical modification conditions of the electrode were the electrodeposition of AuNPs for 240 s, Protein A immobilization for 30 min, and the incubation time of H9A and VP1 for 45 min.

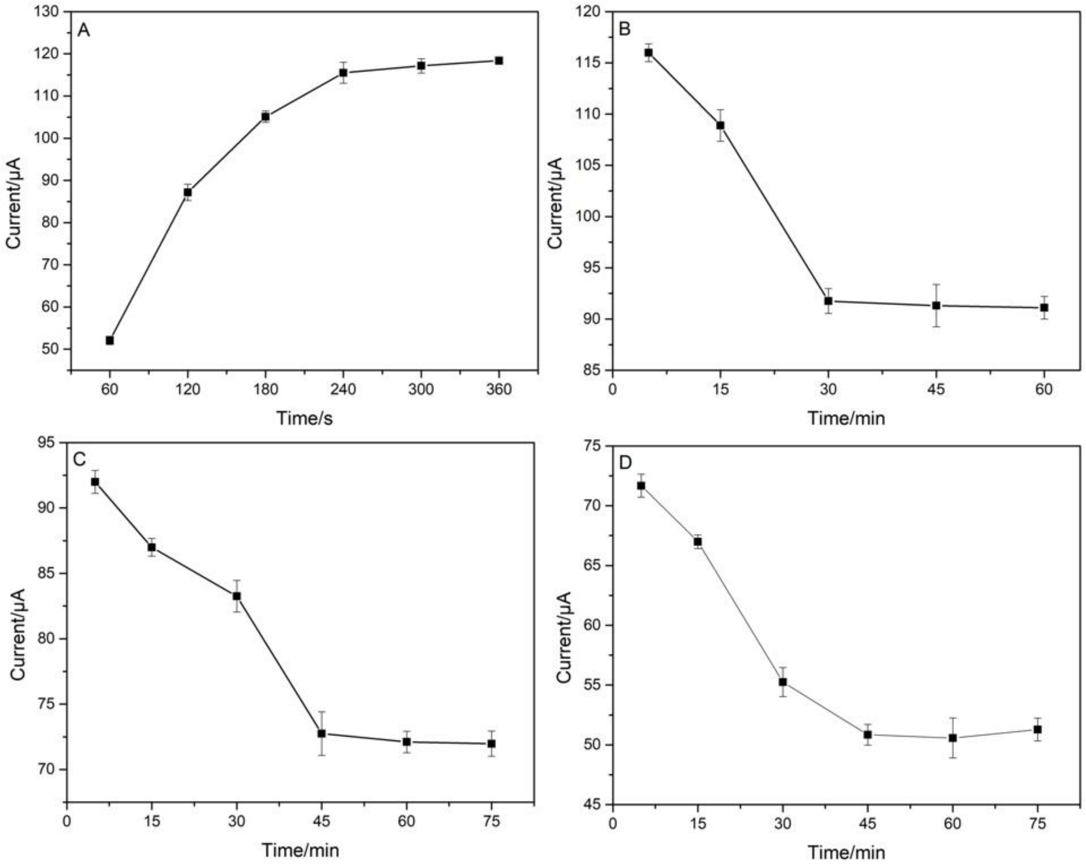

Figure S1 Optimization of the modification conditions for the electrochemical biosensors. **(A)** electrodeposition time of AuNPs; **(B)** protein A immobilization time; **(C)** Incubation time of H9E; **(D)** Incubation time of VP1.
